# Children's Fears

**Published:** 1920-04-10

**Authors:** 


### THE PUBLIC WELL-BEING.

### CHILDREN'S FEARS.

Child-study has become a science now, and a science sometimes too rigid for the elusive sensibility of many child-minds. It is almost in danger of committing in this direction such errors as used to be committed in the other direction. Acute imagination and a certain kind of sympathetic understanding are necessary—understanding like that shown at its best in those delightful essays on childhood by Mrs. Meynell, or in the perfect children's verses of Mr. Walter de la Mare. It is, in fact, poetic imagination that one needs as much as anything. One must believe in fairies, so to speak, and in hob-goblins too. There is one way in which the prosaic and unpoetic progress of science seems of itself to have destroyed one of the greatest sources of terror in children. Where are the bogies and horrors of the childhood of fifty years ago? One has a theory (writes a correspondent) that they have largely disappeared, that children of to-day have less fear of all the nameless terrors of the dark, because of the change from the flickering shadows of the candle to the steady bright light of electricity. Lovers of children will be thankful if this is so, and if the little ones of to-day escape such terrifying nights as the writer experienced when, with her sisters at a boarding-school, she had no companions anywhere within hearing of her on the top floor of the house. If anyone of the kind trio who bade that child good-night had imagined the horrors they were inflicting, they themselves would have been horrified. A sensitive child will often go through anything rather than admit such fears.

One of Miss Charlotte Yonge's stories has the following passage:—

"Bed-time came at last—horrible bed-time, with all its terrors! At first Kate persuaded Josephine and her light to stay till sleep came to put an end to them; but Lady Barbara came up one evening, declared that a girl of eleven years old must not be permitted in such childish nonsense, and ordered Josephine to go down at once and always to put out the candle as soon as Kate was in bed.

"Lady Barbara would hardly have done so if she had known how much suffering she caused, but she had always been too sensible to know what the miseries of fancy could be, or how the silly little brain imagined everything possible and impossible."

Here, again, it is not the tiny child who is the terrified one, but a child thought generally to be past the age of such fears. At the same time, there are, without doubt, many ignorant parents and attendants of children who are still so lacking in imagination or regard for their children's welfare that one form of attempting to secure discipline is a deliberate threat of danger. Some of the cruder examples of this are "the dog will eat you up if you are not good," or "the horse will come after you if you are naughty," or what is a worse form of this foolishness, some similar reference to what exists only in the child's mind, such as the "bogey-man."

The truth is that the proper spiritual, physical, and mental upbringing of children requires such instinctive good sense and imagination, positively exercised, that one wonders how many children are given an all-round chance. Physical welfare is very important, but spiritual welfare, in the broadest meaning of the term, must not be overlooked. It may not prevent rickets; it may be more difficult than the prevention of rickets; but as a tonic influence on the child's spirit, and, very directly through that, on the child's health, its importance is beyond computation. A child is a sensitive flower freshened by the early morning dews and warmed by the clear sunshine, but often the prey of blasting winds, of the rough-handed gardener, or of those who are not gardeners at all. The folk for whom maternity and infant welfare workers endeavour to do all they can are not the only ones who lack knowledge and understanding, and the worst of it is that these others think they know.

